# Application of a Terahertz System Combined with an X-Shaped Metamaterial Microfluidic Cartridge

**DOI:** 10.3390/mi11010074

**Published:** 2020-01-09

**Authors:** Shih-Ting Huang, Shen-Fu Hsu, Kai-Yuan Tang, Ta-Jen Yen, Da-Jeng Yao

**Affiliations:** 1 Department of Power Mechanical Engineering, National Tsing Hua University, Hsinchu 30013, Taiwan; doublu1208@gmail.com; 2 ACE BIOTEK Co. Ltd., Hsinchu 30261, Taiwan; steve_hsu@acesolution.com.tw (S.-F.H.); david_tang@acebiotek.com (K.-Y.T.); 3 Department of Material Science Engineering, National Tsing Hua University, Hsinchu 30013, Taiwan; tjyen@mx.nthu.edu.tw; 4 Institute of NanoEngineering and MicroSystems, National Tsing Hua University, Hsinchu 30013, Taiwan

**Keywords:** terahertz radiation, metamaterials, microfluidics

## Abstract

Terahertz (THz) radiation has attracted wide attention for its ability to sense molecular structure and chemical matter because of a label-free molecular fingerprint and nondestructive properties. When it comes to molecular recognition with terahertz radiation, our attention goes first towards the absorption spectrum, which is beyond the far infrared region. To enhance the sensitivity for similar species, however, it is necessary to apply an artificially designed metamaterial sensor for detection, which confines an electromagnetic field in an extremely sub-wavelength space and hence receives an electromagnetic response through resonance. Once the resonance is caused through the interaction between the THz radiation and the metamaterial, a minute variation might be observed in the frequency domain. For a geometric structure of a metamaterial, a novel design called an X-shaped plasmonic sensor (XPS) can create a quadrupole resonance and lead to sensitivity greater than in the dipole mode. A microfluidic system is able to consume reagents in small volumes for detection, to diminish noise from the environment, and to concentrate the sample into detection spots. A microfluidic device integrated with an X-shaped plasmonic sensor might thus achieve an effective and highly sensitive detection cartridge. Our tests involved not only measurements of liquid samples, but also the performance of a dry bio-sample coated on an XPS.

## 1. Introduction

The terahertz (THz) gap normally refers to a frequency band ranging from 0.1 to 10.0 THz, which is located between the microwave and infrared regions of the electromagnetic spectrum [1]. Compared with electromagnetic waves in other ranges, terahertz radiation is able to penetrate, without destruction, most non-polar materials in a way that is similar to a property of microwaves, and can be applied to security tests because of its ability to discern objects [2]. In addition, terahertz radiation might be readily absorbed by polar materials, such as water molecules and ammonia molecules, for which the characteristics of vibrational and rotational transitions all occur in the THz frequency band [3,4]; these molecular structures consequently generate a different mode of resonance, change of refractive index and absorbance, and present distinct spectral characteristics. This radiation can, therefore, be useful to identify various biochemical structures as weak interactions, including hydrogen bonds and van der Waals, and the low-frequency vibration and rotation of biomolecules can be probed by THz spectra [5]. Based on these properties, applications arise in the biomedical field, such as for disease detection, that can be distinguished according to the molecular structure of the antigen corresponding to the type of disease through its identification in the THz spectrum [6]. For the detection of cancer, cancer cells contain much more water than ordinary cells, which can be identified in a THz image with rapid screening because of the sensitivity to polar molecules [7].

A metamaterial is composed of various materials with a designed geometric structure, which exhibits specific properties of relative permittivity and permeability that differ from those properties of ordinary materials. When interacting with electromagnetic waves, a periodically arranged conductive element can be an effective electromagnetic scattering medium under a condition where the wavelength far exceeds the distance between the elements [8]. That is, a metamaterial is able to produce an effective negative permeability, and a negative permittivity that is lacking in ordinary materials [9,10]. Once it interacts with electromagnetic waves, a frequency resonance with a high quality factor is formed, which is a physical phenomenon generating asymmetric linear resonances [11]. Such a resonance causes a strong absorption effect—a resonant dip can be observed with an abrupt decrease of signal strength that serves as a pointer in the frequency domain. When the dielectric environment alters, a signal can be obtained through the dip offset. Thus, a combination of THz radiation and a metamaterial can likely increase the inherent sensitivity of THz radiation as well as achieve label-free detection making it worthy of application in the biomedical field in the future.

As the development of technologies—such as in the semiconductor electronics industry and for a microelectromechanical system (MEMS)—becomes increasingly mature, the size of the lithographic process is decreased to a micrometer and even a nanometer scale, which has led to the development of microfluidics. The concept of a micro total analysis system (µTAS), which was proposed to emphasize a microfluidic device produced with photolithographic technology, can demonstrate multistep biochemical experiments with decreased consumption of reagents and a rapid reaction to achieve a high efficiency—these advantages of miniaturization and the integration of genetic analysis were mentioned by Manz et al. in 1990 [12]. The modest consumption of reagents and the decreased size of instruments can be interpreted to combine the various experimental processes in a traditional biomedical laboratory, such as solution mixing, dilution, material separation, extraction, and detection into a miniature chip, and give rise to lab-on-a-chip (LOC) [13].

The detection of biological samples, such as antibody antigens, fungal cells, and biological proteins, formerly applied a polymerase chain reaction (PCR) and fluorescence-based microbial detection system, which is generally considered to be an effective way to implement visible tracking and monitoring of biomolecular compounds [14,15], but these complicated methods have disadvantages of being time-consuming, expensive, and requiring label-intensive detection. With improved laser and semiconductor techniques, THz sensing technology has definitely become a potential method of biomedical detection because of its benefits of high effectiveness, sensitivity, and non-invasive detection [16,17]. With the sensing ability of a THz system, combining the effect of a metamaterial and a microfluidic system into a detection cartridge is a promising idea with which to achieve a goal of rapid, label-free, and non-destructive detection.

## 2. Materials and Methods

### 2.1. Design of a Metamaterial

**Figure 1:**
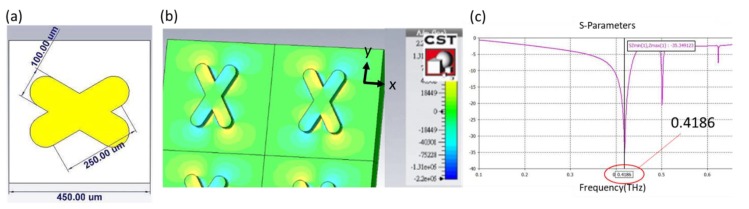
(**a**) Scale of an XPS, of linewidth 100 μm, length 250 μm and period 450 μm. (**b**) x-component of electric field distribution in a quadrupole resonance. (**c**) A simulation result shows a resonant dip at frequency 0.4186 THz.

Plasmonic sensors are known to be sensitive to the change of the refractive index of an environment. When THz radiation is combined with an artificially designed metamaterial, as presented in this research, the quadrupole resonant mode of an X-shaped plasmonic sensor (XPS) can create a quadrupole resonance [18], cause a cancellation of reflection, lead to high absorption, and achieve a high quality factor and great sensitivity. The XPS structure with the angle orientation of the two arms is 60 or 120 degrees, which is based on a CST simulation, has a linewidth of 100 μm, length of 250 μm, and period of 450 μm, as shown in Figure 1a. In Figure 1b, the results are shown of the metamaterial with Si substrate, and the permittivity of it is acquired from the refractive index(n_Si_ = 3.38), which was taken from the reference [19]. When the incident THz wave is controlled in x-component polarization, the x-component of the electric field distribution exhibits positive and negative values on two opposite bars that contribute to the quadrupole resonant mode of an XPS; the resonant dip occurs at 0.4186 THz, as shown in Figure 1c. To define the sensor sensitivity and the variation in refractive index of the environment, the spectrum presents a narrow resonant dip that implies a large figure of merit (FOM) and quality factor [20]. With semi-quantitative analysis [21], applying a metamaterial as a sensing chip is hence the fundamental core of detecting a change of permittivity in the environment.

### 2.2. Microfluidic System

For a future application of a detection cartridge, which will definitely tend to become used in the biomedical field, it is important to select an appropriate material for the microfluidic layer. For this purpose, a highly biocompatible polymer called polydimethylsiloxane (PDMS) has been widely applied to a LOC because of its benefit of modest cost and simple fabrication. However, PDMS is a normal material in liquid form deployed in varied proportions that depend on the particular experiment; the mold structure should thus be fabricated before molding the PDMS microfluidic structure. In general, these processes become achievable through photolithography using a SU8 photoresist; the channel height is readily defined according to the parameters used in fabrication.

### 2.3. Fabrication of a Detection Cartridge

The detection cartridge, which is composed of a metamaterial sensor and a microfluidic system, is fabricated with photolithography. It can be divided into two layers; the bottom layer is XPS and the upper layer is a PDMS microfluidic structure.

For the bottom layer that begins at the silicon substrate, it is first necessary to clean the wafer using a standard process—HMDS is evaporated for 5 min to enhance the adhesion of the photoresist. Second, a positive photoresist (AZ5214) is spin-coated at 3000 rpm for 30 s; soft baking is carried out at 100 °C for 1 min. Third, exposure and deployment follow, in which the exposure is undertaken at energy density 60 mJ/cm^2^. Fourth, after the development, e-beam evaporation proceeds to deposit gold of thickness 200 nm with an adhesion layer of 20 nm. Finally, the lift-off process removes superfluous matter other than the XPS patterns.

For the upper layer, the SU8 mold begins from a silicon substrate; the experiment is demonstrated for a channel of height 35–40 μm. First, after a standard cleaning process, negative photoresist SU8 was directly coated at 500 rpm pre-spin for 10 s with spinning at 3500 rpm for 30 s. Second, soft baking began at 75 °C, and increased 5 °C every 3 min until 95 °C and was maintained for 15 min, which avoids the SU8 becoming heated too rapidly and cracking. Third, in the exposure and deployment, the exposure proceeds at energy density 250 mJ/cm^2^. Finally, hard baking for 10 min strengthens the SU8 structure. After completing the SU8 mold, the deployment of PDMS (Sil-More Industrial Ltd., Taipei, Taiwan) was controlled the ratio that A:B agent = 10:1; pouring into the SU8 mold was followed by heating at 80 °C for 40 min resulting in a 2 mm thickness of PDMS layer. Eventually, once the PDMS layer is confirmed to be completely solidified, it should be easily peeled off.

**Figure 2:**
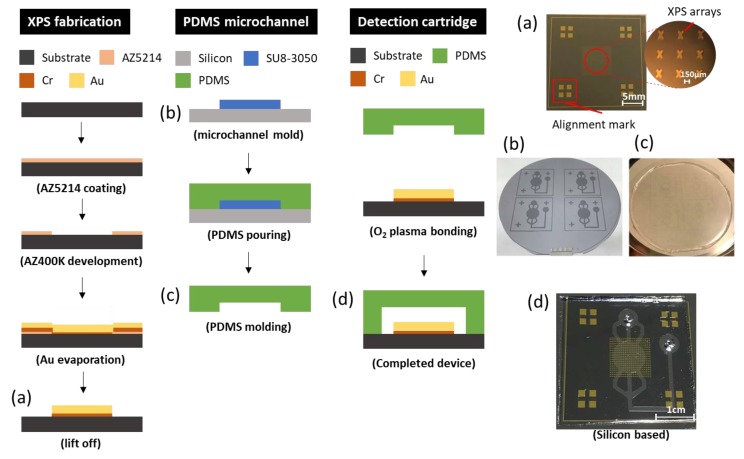
Fabrication: (**a**) XPS metamaterial; (**b**) SU8 mold; (**c**) PDMS microfluidic layer; (**d**) finished product (detection cartridge).

After the bottom and the upper layers are ready, the final step is to combine these into a detection cartridge. To achieve bonding energy, hydrophobic properties must be transformed into hydrophilic at the interface through a surface modification employing an oxygen plasma. If this modification is successful, the final product is completed on aligning two layers as soon as possible and baking at 65 °C for 5 min. Figure 2 shows the entire process including the upper and bottom layers and the bonding technique.

### 2.4. Experimental Setup

**Figure 3:**
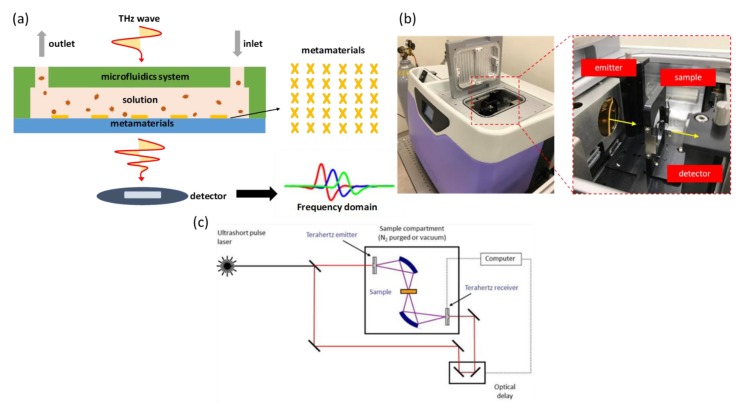
(**a**) Illustration of detection mechanism; the THz radiation penetrates the entire cartridge. (**b**) Internal setup in the chamber (TeraPulse 4000). (**c**) Setup of optical components inside the equipment.

The detection mechanism proceeds with a transmission measurement through the detection cartridge, which is illustrated in Figure 3a. When pulsed, THz radiation penetrates the detection cartridge—once the sample inside the cartridge exhibits a difference of permittivity, the response signal in the time domain received by the detector can detect the variation of a resonant dip after post-processing the signal in the frequency domain. 

The equipment for detection using THz time-domain spectroscopy (THz-TDS) is called a TeraPulse 4000 (product of TeraView, UK). The generation of terahertz pulsed radiation is based on a photoconductive switch in which terahertz photoconductive emitters rely on the production of few-cycle terahertz pulses using a femtosecond laser to excite a biased gallium arsenide antenna. This technique is inherently broadband, with the emitted power distributed over a frequency range from 60 GHz to 4 THz (wavenumber 2–133 cm^−1^). When demonstrating the measurement, the detection cartridge is installed at the fixed sample position in the chamber, as shown in Figure 3b. After setting up, the chamber is sealed and kept filled with dry air to eliminate any influence of water vapor. The spectrum is collected at a rate 30 s^−1^; spectral resolution 32 GHz allows rates of data acquisition up to 50 Hz. The internal optical components are illustrated in Figure 3c.

### 2.5. Effect of XPS in an Actual Measurement

**Figure 4:**
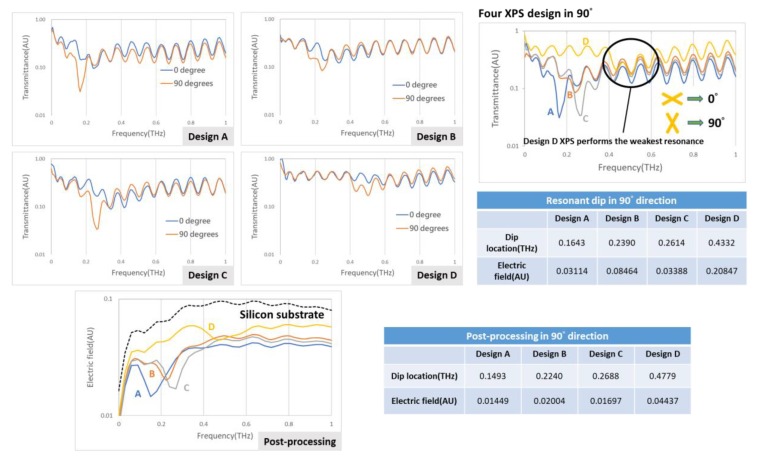
Effect of four XPS designs in an actual situation; all perform best in direction 90°.

Before attempting detection using the cartridge, the first consideration is whether the XPS works in an actual situation; the idea to confirm the effect of XPS hence includes two ways—one is to examine the resonant dip effect of the XPS design on four scales, the other is to vary the direction of the XPS and to conduct each measurement. There are four XPS designs of linewidth 100 μm, length 250 μm, and period 450 μm. In Figure 4, the results show the effect of four XPS designs—A, B, C, and D, which have respective linewidths of 100, 80, 70, and 24 μm; lengths of 250, 162, 150, and 80 μm; and periods of 450, 380, 300, and 200 μm, but they all perform better in direction 90°. On comparing all designs in this direction, these measured spectrums were normalized to a silicon substrate only measurement, however design D displayed the weakest resonant function. The post-processing signals, which were aimed at eliminating the interference in Si substrate, are shown below in the same figure, and the black dashed line indicates a signal of pure silicon substrate revealing removal of the ringing effect. All in all, the evidence is that XPS works in an actual situation and the proper design also finds the right direction.

**Figure 5:**
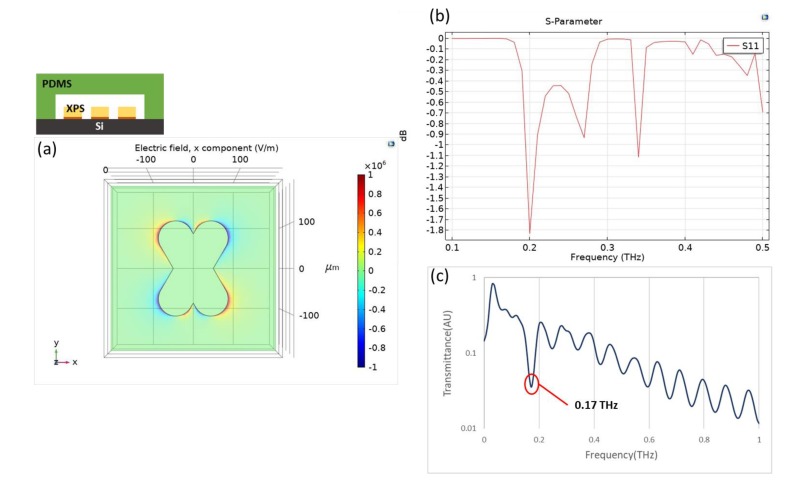
Simulation for microfluidic cartridge. (**a**) X-component of electric field distribution in a quadrupole resonance. (**b**) A simulation result shows a resonant dip at 0.20 THz. (**c**) An experimental result shows a resonant dip at 0.17 THz.

Considering the application of a microfluidic cartridge, it is necessary to simulate for the device with multilayers including PDMS, silicon substrate, and gold where the thickness is 2 mm, 500 μm, and 200 nm, respectively. The XPS in design B is conducted in the structure that the following measurements are related to it, based on COMSOL simulation [22]. Similarly, the microfluidic cartridge performs a quadrupole resonant effect and the resonance occurs at 0.20 THz, as shown in Figure 5a,b. The experimental result for the same structure is shown in Figure 5c and its resonance is located at 0.17 THz, which is close to the simulation results.

### 2.6. Introduction of Sample for Demonstrations

Our trial of the detection cartridge included several demonstrations that involved varied concentrations of IPA and glucose solutions, concurrently examining the influence of water absorption. In the measurements of IPA solution, the controlling concentrations were 100%, 75%, 50%, 25%, and 0% IPA; the same cartridge was applied and ten times the volume in the channel was injected before transforming to the next concentration. In the measurement of the glucose solutions, the fixed concentrations ranged from 0.1% to 0.5%, which correlates with the application to detect diabetes disease. We tested dry samples as well; the detected objects were lung cancer and breast cancer cells at concentration of 10^7^ cells per mL.

## 3. Results

### 3.1. Inspection of Water Absorption

**Figure 6:**
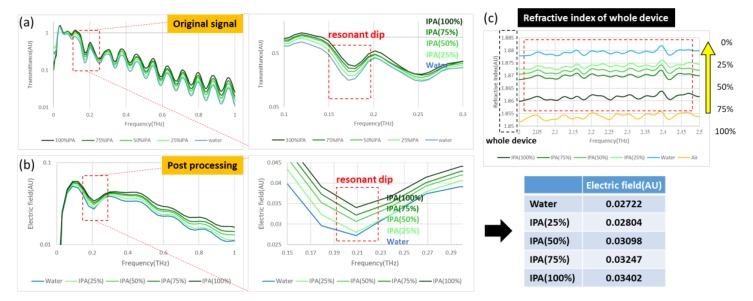
Spectrum of IPA solution at five concentrations: (**a**) Original signal with fluctuation; (**b**) Post-processing signal to eliminate interference; (**c**) Refractive index of the entire device, the value increases as the water ratio rises.

To observe the absorption of water in the THz regime, even if the trial is under a microfluidic cartridge, we measured for test purposes the IPA solution of varied concentrations—100%, 75%, 50%, 25%, and 0%—the XPS of design B was adopted for this experiment. The results appear in Figure 6. The first-hand information in the sample spectrum displays an intense fluctuation because of interference from silicon, which seriously affects the observation of a resonant dip, as presented in Figure 6a. Hence, it is necessary to operate on the post-processing signal to eliminate the impact of silicon, as shown in Figure 6b. When the effect of this interference is removed, all signals exhibit clearly a gradient variation of the IPA solution at varied concentrations. As a consequence, when attention is focused on the resonant area, the strength of the electric field decreases as the IPA concentration decreases, because the water absorption is greater than that of IPA. Analysis of the refractive index, shown in Figure 6c, which displays the value of the entire microfluidic cartridge, indicates that this index increases as the IPA concentration decreases, opposite to the result in Figure 6b. The absorption of water observed in the frequency domain is thus caused by the refractive index of water in the THz regime.

### 3.2. Trial for Diabetes Decease Detection

**Figure 7:**
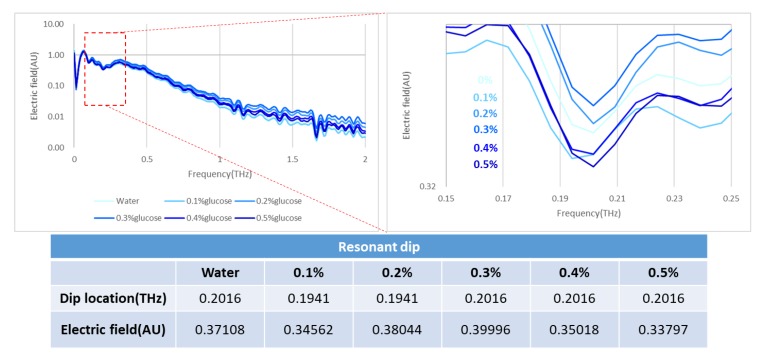
Trial of measurement of glucose aqueous solution from 0% to 0.5% with no tendency of variation.

According to American Diabetes Association (ADA), the mechanism of diabetes detection refers to 70–99 mg/dL and 80–130 mg/dL for the level of blood sugar, which belongs to a normal person without diabetes and diabetics under a fasting condition, respectively. Their ranges are equivalent to 0.41–0.56% glucose solution. To achieve the detection of diabetes using the microfluidic cartridge, one can simulate a similar condition with a glucose aqueous solution from 0.1% to 0.5%. The result shown in Figure 7 indicates a tendency for the frequency resonance to vary with the changing concentration of the glucose solution. The experiment was repeated more than five times, but all observations were unsuccessful; the metamaterial effect was probably influenced by water absorption.

### 3.3. Detection of Cancer Cells

**Figure 8:**
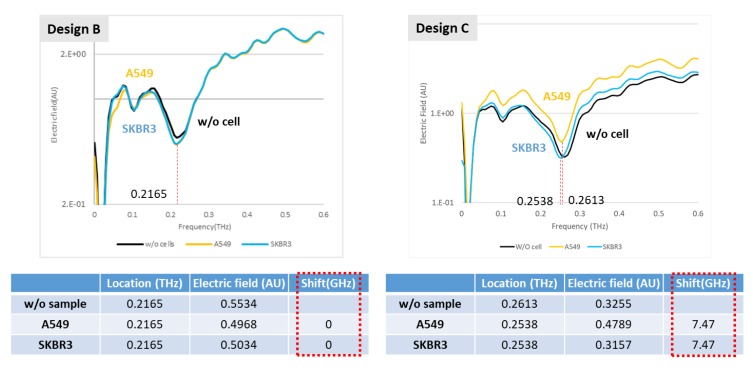
Detection of cancer cell on XPS of two designs; the red shift has extent 7.47 GHz in design C for both cells.

For most biomedical applications, bio-samples tend to be stored with a buffer; this buffer with the solvent generally contains water in a large proportion that is definitely an obstacle of detection in the THz regime; hence, the channel height of the microfluidic cartridge must be decreased as much as practicable for the detection of an aqueous bio-sample. Before attending to the goal of a wide application to a microfluidic cartridge, we undertook an experiment to differentiate cancer cells in dry conditions of two types, lung and breast cancers. Without a microfluidic system, the samples were directly coated on the XPS metamaterial as a fixed amount; the effect was observed in designs B and C. As Figure 8 shows, in which A549 and SKBR3 represent the lung cancer cells and breast cancer cells respectively, a clear frequency resonant effect is exhibited in both XPS designs, which distinguishes the two cell types according to the frequency spectrum. For the XPS in design C, the recognition improved because of the different location of the resonant dip that both cells exhibited, with a red shift relative to no cells coated on the XPS, favorably recording a different spectrum for the two cell types. For the different effect on XPS in the two designs, this evidence indicates that the same species display varied features at different frequency.

## 4. Conclusions

A novel idea to integrate a metamaterial and a microfluidic system into a detection cartridge is undoubtedly an effective application in the biomedical field. Accordingly, this research demonstrates trials of aqueous solutions, such as IPA and glucose, which show that water absorption has an enormous influence on the detection mechanism. This influence of water absorption in the THz regime was far more than expected on applying a microfluidic system; the thickness of the channel should apparently be further decreased, even to a nanometer scale, so that the effect of water can be removed in the cartridge. The second-best operation demonstrated the detection of cancer cells without a microfluidic system, providing strong evidence of an ability to distinguish two cell types through the XPS metamaterial in two designs. Eventually, a reliable metamaterial design will be able to fulfill the applications of a microfluidic cartridge with an enhanced channel design.
